# Machine-learning-informed scattering correlation analysis of sheared colloids

**DOI:** 10.1107/S1600576725003280

**Published:** 2025-05-31

**Authors:** Lijie Ding, Yihao Chen, Changwoo Do

**Affiliations:** a Neutron Scattering Division Oak Ridge National Laboratory,Oak Ridge TN37831 USA; b Department of Physics and Astronomy, University of Pennsylvania, Philadelphia, PA19104, USA; Australian Centre for Neutron Scattering, ANSTO, Australia

**Keywords:** small-angle scattering, machine learning, Gaussian process regression, Monte Carlo simulations, colloids

## Abstract

We have carried out theoretical analysis, Monte Carlo simulations and machine-learning analysis to quantify microscopic rearrangements of dilute dispersions of spherical colloidal particles from coherent scattering intensity. Both monodisperse and polydisperse dispersions of colloids were created and underwent a rearrangement consisting of an affine simple shear and non-affine rearrangement using the Monte Carlo method. We calculated the coherent scattering intensity of the dispersions and the correlation function of intensity before and after the rearrangement and generated a large data set of angular correlation functions for varying system parameters, including number density, polydispersity, shear strain and non-affine rearrangement. Singular value decomposition of the data set shows the feasibility of machine-learning inversion from the correlation function for the polydispersity, shear strain and non-affine rearrangement using only three parameters. A Gaussian process regressor is then trained on the data set and can retrieve the affine shear strain, non-affine rearrangement and polydispersity with relative errors of 3%, 1% and 6%, respectively. Altogether, our model provides a framework for quantitative studies of both steady and non-steady microscopic dynamics of colloidal dispersions using coherent scattering methods.

## Abstract 2

We have used machine-learning-informed scattering correlation analysis to extract polydispersity and microscopic rearrangements from scattering data, enabling precise insights into dynamic processes in colloidal dispersions

## 1. Introduction

Quantification of the microscopic dynamics of materials made of nanometre- to micrometre-scale constituents is vital in understanding the origins of macroscopic mechanical properties and designing novel functional materials for pharmaceutical, environmental and other industrial applications (Wu *et al.*, 2020 ▸). While traditional optical microscopy can provide real space information, it is limited by its resolution and the opacity of the materials (Badon *et al.*, 2017 ▸). Scattering techniques (Murphy *et al.*, 2020 ▸; Guinier *et al.*, 1955 ▸) like X-ray photon correlation spectroscopy (Chu & Hsiao, 2001 ▸; Shpyrko, 2014 ▸; Leheny *et al.*, 2015 ▸; Madsen *et al.*, 2020 ▸), dynamic light scattering (Goldburg, 1999 ▸; Aime & Cipelletti, 2019 ▸) and small-angle neutron scattering (Shibayama, 2011 ▸; Chen, 1986 ▸) provide great opportunities to probe the microscopic information of such materials, and they have been deployed to study the microstructural dynamics of colloidal (Chen *et al.*, 2020*a* ▸; Donley *et al.*, 2023 ▸), polymeric (Ruocco *et al.*, 2013 ▸) and atomic materials (Lüttich *et al.*, 2018 ▸). However, the challenge of scattering techniques is the quantification of microscopic rearrangement in real space back from the scattering patterns (Fourier space) which, most of the time, are only available in a limited range of two dimensions (in the case of an area detector) and sometimes even one dimension (in the case of a photon-counting device). Previous efforts have investigated steady shear (Burghardt *et al.*, 2012 ▸), diffusion (Leitner *et al.*, 2009 ▸), localization of particles (Chen *et al.*, 2020*b* ▸) *etc.* Most of the schemes characterize the temporal correlation function of the scattering intensity and require an average of the intensity or/and the correlation function of the intensity in a time interval assuming steady dynamics. On the other hand, non-steady microscopic dynamics, like non-affine rearrangement, are widely observed in real space and play an important role in the non-linear properties of soft materials (Keim & Arratia, 2015 ▸; Wen *et al.*, 2012 ▸), like yield (Jana & Pastewka, 2019 ▸) and memory effects (Galloway *et al.*, 2022 ▸). However, they are much less studied in coherent scattering experiments due to their non-linear and transient nature, and their interpretation often requires intensive modeling and computation (Ma *et al.*, 2014 ▸; Banetta *et al.*, 2022 ▸; He *et al.*, 2024 ▸).

To address these issues, we developed a generalized theoretical and machine-learning (ML) framework (Murphy, 2012 ▸; Carleo *et al.*, 2019 ▸) to quantify affine and non-affine rearrangements of dilute, both monodisperse and polydisperse, colloidal dispersions using the correlation function of coherent scattering intensity. Recently, various ML frameworks have been developed to interpret scattering experiments, such as SCAN [SCattering Ai aNalysis (Tomaszewski *et al.*, 2021 ▸)], which automates structural analysis using predefined particle shape models, and CREASE [computational reverse-engineering analysis for scattering experiments (Anker *et al.*, 2023 ▸; Lu & Jayaraman, 2024 ▸; Akepati *et al.*, 2024 ▸; Heil *et al.*, 2023 ▸; Wu & Jayaraman, 2022 ▸; Wessels & Jayaraman, 2021 ▸)], which leverages genetic algorithms and surrogate ML methods (*e.g.XGBoost*) to reconstruct 3D structural features, including domain size, shape, orientation and spatial distributions from scattering profiles. In particular, CREASE-2D (Akepati *et al.*, 2024 ▸) enabled analysis of 2D scattering patterns, avoiding the traditional approximate analysis of scattering intensity profiles. Other ML methods have also been applied for particle tracking and ordered structures in soft materials (Clegg, 2021 ▸) and surface scattering analysis (Hinderhofer *et al.*, 2023 ▸). Our approach is distinct from these methods in that it directly correlates coherent scattering intensity with physical parameters such as shear strain, non-affine rearrangement and polydispersity in dynamically sheared colloidal systems. Unlike generalizable tools, our ML approach utilizes Monte Carlo (MC) simulation (Krauth, 2006 ▸) to generate particle configurations and rearrangements in two dimensions. The coherent scattering intensity of the particles and the correlation function of the intensity were calculated before and after the rearrangement, and using singular value decomposition (SVD) we extracted three essential features of the correlation function that reliably recover the magnitude of both affine and non-affine rearrangements and polydispersity. Similar approaches have been applied to other soft-matter systems including colloids (Chang *et al.*, 2022 ▸; Huang *et al.*, 2023 ▸; Tung *et al.*, 2022 ▸; Tung *et al.*, 2024*a* ▸), lamellae (Tung *et al.*, 2024*b* ▸; Tung *et al.*, 2025 ▸) and polymers (Tung *et al.*, 2023 ▸; Ding *et al.*, 2024*a* ▸; Ding *et al.*, 2024*b* ▸; Ding *et al.*, 2025 ▸). We then used Gaussian process regression (GPR) (Williams & Rasmussen, 2006 ▸) to map from scattering data to the system parameters including polydispersity, shear strain and non-affine rearrangement. We also tested our trained GPR using simulation data aside from the training data; good agreement between the ML-extracted system parameters and the MC references was achieved, showing the accuracy of our approach. Our model can be easily applied to coherent scattering experiments to extract microscopic rearrangements between two scattering patterns, which is especially useful for studies of non-steady and transient dynamics.

The rest of this paper is organized as follows: Section 2 introduces our colloidal systems, the theoretical analysis of coherent scattering intensity, MC simulation and a brief summary of the GPR; our results are presented in Section 3 to illustrate the scattering intensity and correlation function of the colloidal dispersions under rearrangements, validate the feasibility for ML inversion of system parameters using SVD of the correlation and show the application of ML analysis for scattering data using GPR; finally, we summarize our paper and discuss potential future directions following this work in Section 4.

## 2. Methods

### 2.1. Coherent scattering and rearrangement transformation

The normalized scattering intensity of a polydisperse dispersion of *N* spherical particles with a configuration of 2D coordinates 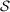 = {(*x*, *y*)} is given by (Chen, 1986 ▸)
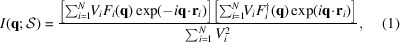
where *V_i_* = (4π/3)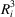 is the volume of particle *i* with radius *R_i_*, **q** is the scattering wavevector and **r**_*i*_ = (*x_i_*, *y_i_*) is the position of particle *i*. *F_i_*(**q**) is the form factor amplitude of the *i*th particle such that (Guinier *et al.*, 1955 ▸)
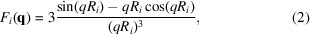
where *q* = |**q**| is the magnitude of the scattering wavevector.

**Figure 1:**
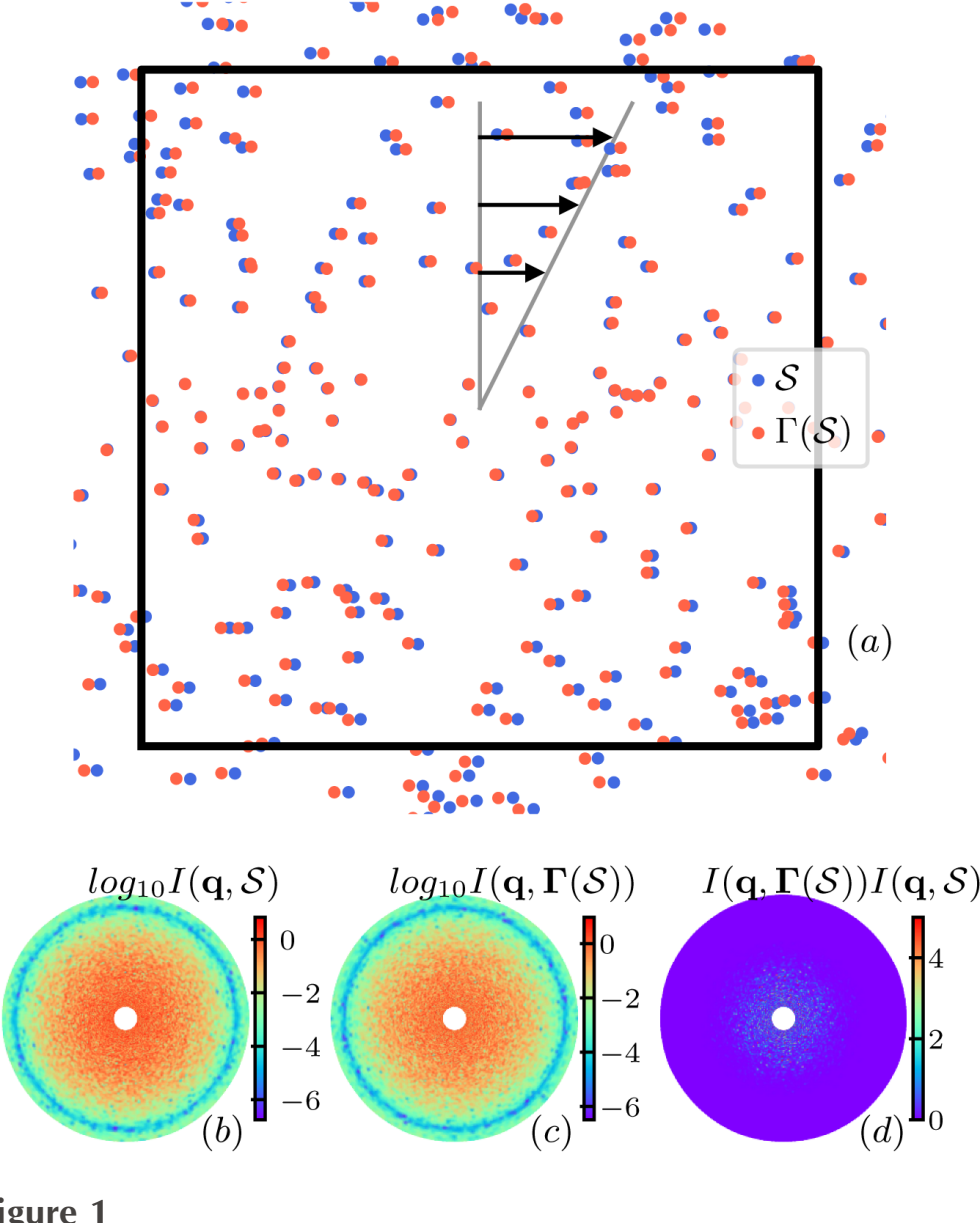
Illustration of a single configuration of particles undergoing transformation Γ, in which (*nL*^2^, *R*_s_, *D*_2_, γ*L*) = (150, 0, 0.5, 30). The black frame indicates the region the beamline shines on. (*a*) Spatial distribution of particles before (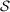) and after [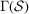] the transformation. For better visualization, the particles are not to scale. Scattering intensity of the configuration (*b*) before and (*c*) after the transformation Γ. (*d*) Product of the correlation function between the scattering intensity of transformed and non-transformed configurations.

The rearrangement transformation of the particle positions consists of an affine simple shear deformation along the *x* axis (shear gradient runs along the *y* axis) with a shear strain γ, as shown in Fig. 1(*a*), and a non-affine rearrangement where the particles are randomly displaced by δ*x_i_* and δ*y_i_*, which follow a Gaussian distribution with zero mean and a standard deviation *D*_2_. Such a transformation Γ is expressed as
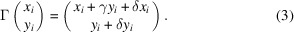


In homodyne scattering, the average translation of all the particles has no effect on the scattering intensity, so we choose the form of equation (3) to have a fixed origin for the affine shear.

The correlation function of two scattering intensities before and after the transformation Γ is 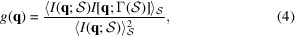
where 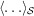 is the average over different realizations of the configuration.

In the case of dilute dispersions of monodisperse spherical particles, including materials doped with a dilute amount of monodisperse tracer particles, the positions of different particles and, thus, their rearrangements are uncorrelated, and the correlation function *g*(**q**) associated with the transformation Γ is predicted as [similar to that in Aime & Cipelletti (2019 ▸)]
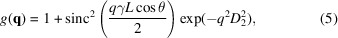
where sinc(*x*) = sin(*x*)/*x*, *L* is the size the scattering beam, and θ is the angle between **q** and the *x* axis.

### 2.2. Monte Carlo simulation

We sampled the positions of particles 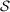 in a [−*L*, *L*]^2^ square with a number density *n*. The radius of the particles follows a log-normal distribution such that 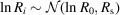, where the polydispersity index of the dispersion, PDI = 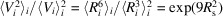, is controlled by *R*_s_ (Kotz *et al.*, 2019 ▸).

We calculated the scattering intensity *I*(**q**) = *I*(*q_x_*, *q_y_*) = 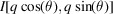 in the polar coordinates for all the particles inside the box of [−0.5*L*, 0.5*L*]^2^ before and after the Γ transformation, and then calculated the correlation function *g*(**q**). The values of *I*(**q**) and *g*(**q**) are averaged over 2 × 10^4^ samples of 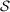 for each set of system parameters (*nL*^2^, *R*_s_, *D*_2_, γ*L*). We also calculated the radial and angular average of the correlation function such that 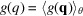 and 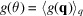, where 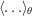 and 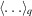 are averages over all measured θ and *q*, respectively. Without loss of generality, we use the natural unit *R*_0_ = 1 for the size of particles and the beam size *L* = 800*R*_0_. We measured *I*(**q**) and *g*(**q**) with respect to 100 different values of 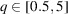 uniformly distributed on a log scale, and 101 different values of 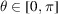 uniformly distributed on a linear scale, and we note that *I*(*q*, θ + π) = *I*(*q*, θ) due to ±**q** symmetry of equation (1). The choice of *R*_0_, *L* and the range of *q* is to mirror experimental conditions: a colloidal dispersion of particles with a radius of 10 nm, a synchrotron X-ray beam size of 8 µm and an area detector at a small-angle scattering setup that covers a wavevector ranging from 0.05 to 0.5 nm^−1^.

### 2.3. Gaussian process regression

To obtain the inverted mapping from the scattering correlation function **x** = *g*(**q**) to the system parameters or inversion targets **y** = (*nL*^2^, *R*_s_, *D*_2_, γ*L*), we trained a GPR using the data generated by MC simulation. In the context of GPR, the prior on the regression function is a Gaussian process, 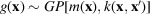, where *m*(**x**) is the prior mean function and *k*(**x**, **x**′) is the covariance function or kernel. The goal of the GPR is to find the optimized posterior 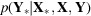 of the function output **y**. The joint distribution of the Gaussian process is (Williams & Rasmussen, 2006 ▸)
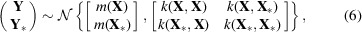
where a constant function is used for the prior mean *m*(**x**), and the kernel consists of a Radial basis function and white noise. 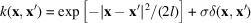, where δ is the Kronecker delta function. *l* and σ denote the hyperparameters corresponding to the correlation length and variance of observational noise, which can be obtained by training on simulation data. In practice, we use the *scikit-learn* Gaussian process library (Pedregosa *et al.*, 2011 ▸) for convenience of implementation and efficiency.

To investigate the distribution of **X**, we define the pair distance distribution function (PDDF) for **X**, 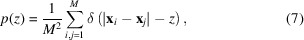
where *M* = |**X**| is the number of data points. In addition, to help determine the appropriate range of hyperparameter *l* when initiating then optimization process, we calculated the autocorrelation function (ACF) for feature μ (Chang *et al.*, 2022 ▸),
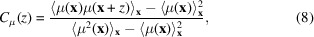
where the 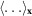 is averaged over all data points in **X**.

## 3. Results

### 3.1. Scattering function of the dispersion

Fig. 1 ▸(*a*) shows an example of the configuration 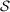 of monodisperse particles before and after the rearrangement transformation Γ, where γ*L* = 30 and *D*_2_ = 0.5. Figs. 1 ▸(*b*) and 1 ▸(*c*) show the corresponding coherent scattering patterns of configurations 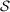 and 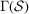, respectively. Speckles are clearly observed in the scattering patterns. Fig. 1 ▸(*d*) shows the product of the two instances of scattering intensity shown in Figs. 1 ▸(*b*) and 1 ▸(*c*).

**Figure 2:**
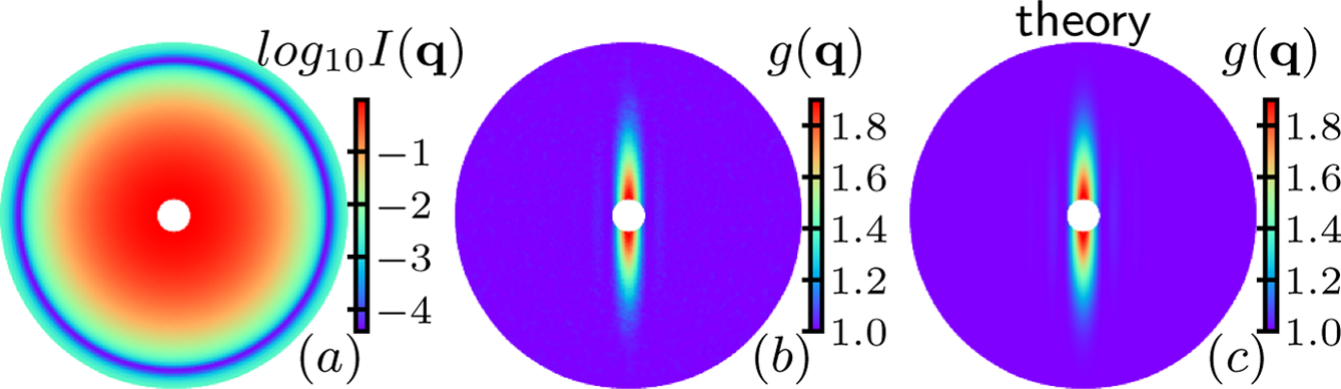
Scattering function *I*(**q**) and scattering correlation function *g*(**q**) of the particles with (*nL*^2^, *R*_s_, *D*_2_, γ*L*) = (150, 0, 0.5, 10). The range of the scattering wavevector is 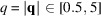, plotted in linear scale. (*a*) Averaged scattering intensity. (*b*) Averaged correlation function. (*c*) Theoretical predicted correlation function for the monodisperse system as in equation (5).

Fig. 2 ▸(*a*) shows an example of the scattering pattern *I*(**q**) of monodisperse particles after averaging over 20000 configurations of 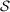. The scattering intensity *I*(**q**) is isotropic and reflects the form factor *F*^2^(**q**) of the spherical particles. Fig. 2 ▸(*b*) shows the correlation *g*(**q**) averaged over 20000 samples. *g*(**q**) is highly anisotropic with a high correlation in the *y* axis (θ = π/2). The non-affine rearrangement is isotropic and causes the decay of structural correlation in all directions. For the affine shear rearrangement [equation (3)], the *y* coordinates of the particles remain unchanged, so the affine shear only leads to the decay of correlation in other directions except the *y* axis (gradient of the affine shear). The pattern of *g*(**q**) is captured very well by the theoretical prediction of equation (5) as shown in Fig. 2 ▸(*c*), and the mean percentage error between the MC calculation *g*(**q**) and the theoretical prediction *g*_theo_(**q**) is Err = 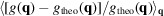 = 0.9%, where 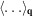 is the average over all **q**.

**Figure 3:**
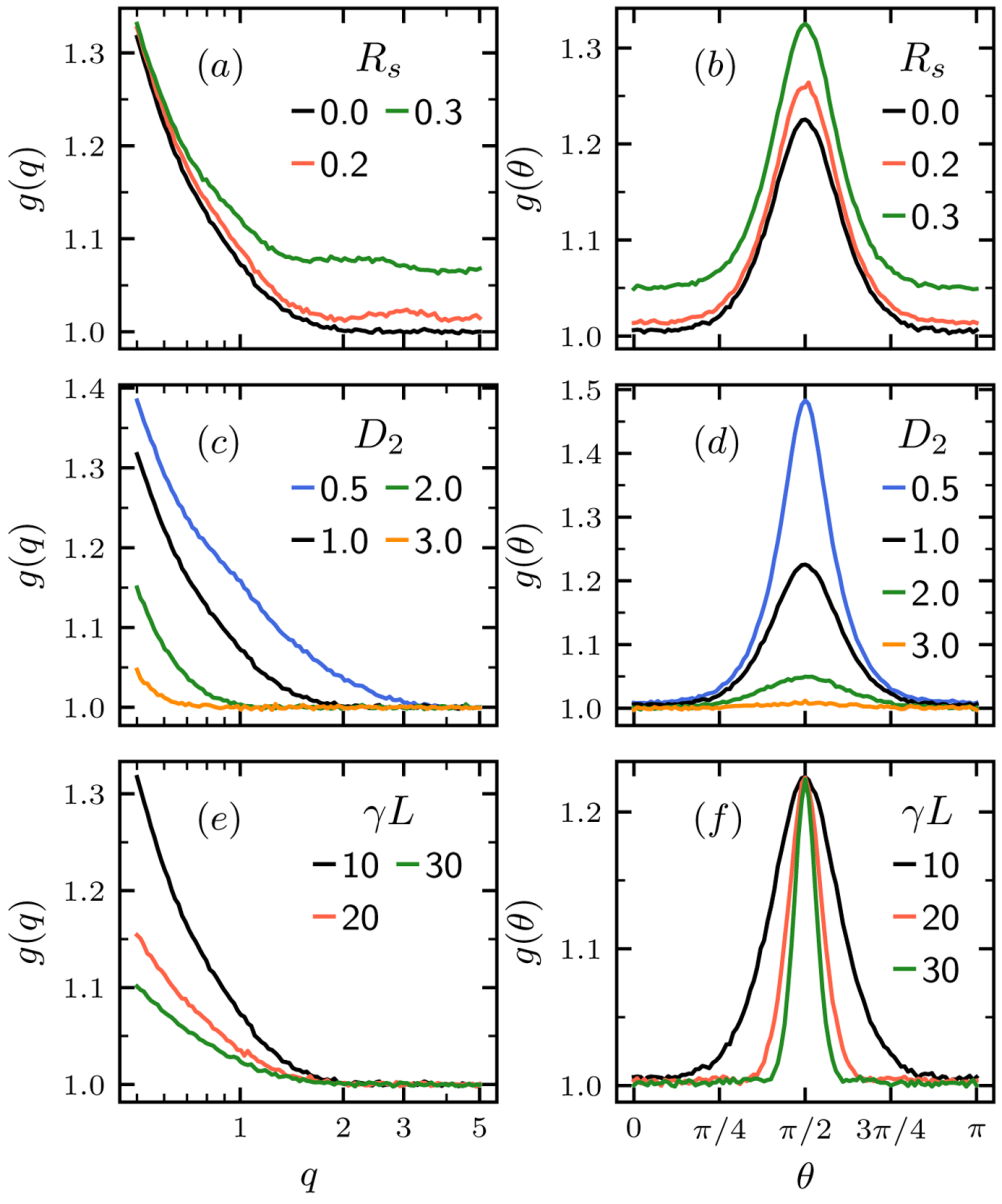
Radial (left column) and angular (right column) correlation functions, *g*(*q*) and *g*(θ), for various polydispersity *R*_s_, non-affine rearrangement *D*_2_ and affine shear γ*L* with a reference of (*n*, *R*_s_, *D*_2_, γ*L*) = (150, 0, 1, 10) represented by the black lines. (*a*) and (*b*) Variable *R*_s_, (*c*) and (*d*) variable *D*_2_, and (*e*) and (*f*) variable γ*L*.

To further quantify the effect of polydispersity, shear strain and non-affine transformation on the correlation function, we used (*R*_s_, *D*_2_, γ*L*) = (0, 1, 10) as a baseline to demonstrate the effect of the three system parameters (*R*_s_, *D*_2_, γ*L*) on *g*(*q*) and *g*(θ). Fig. 3 ▸ shows the radial and angular averaged correlation function *g*(*q*) and *g*(θ) with different values of (*R*_s_, *D*_2_, γ*L*), which alter the correlation function in different ways. The radial correlation function, *g*(*q*), peaks at the lowest *q* and decays at higher *q*. The variation of particle size *R*_s_ controls the height of the plateau of *g*(*q*) at the high-*q* limit, which increases with larger *R*_s_. Affine shear strain γ*L* and non-affine rearrangement *D*_2_ affect the peak of *g*(*q*) at the lowest *q* in a similar way, where increasing both shear strain γ*L* and *D*_2_ lowers the peak. The effect of the three system parameters on the angular correlation function *g*(θ) is more distinguishable, as shown in Figs. 3 ▸(*b*), 3 ▸(*d*) and 3 ▸(*f*). The angular correlation function *g*(θ) has a peak at θ = π/2 (affine shear gradient direction), and γ*L*, *D*_2_ and *R*_s_ affect the width, height and baseline of the peak. Therefore, we focus on the angular correlation function for the rest of the work. However, radial correlation functions also show significant enough feature differences, which can be used for the same inversion analysis presented here.

### 3.2. Feasibility of machine-learning inversion

We generated a data set of 6000 angular scattering correlation functions, **F** = {*g*(θ)}, whose corresponding system parameters **Y** = {(*nL*^2^, *R*_s_, *D*_2_, γ*L*)} are randomly distributed such that 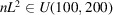, 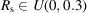, 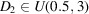 and 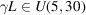, where *U*(*a*, *b*) is a uniform distribution in the interval [*a*, *b*]. Note that *g*(θ) is measured for 101 θ uniformly in [0, π], so **F** is a 6000 × 101 matrix. Following a similar framework to the literature (Chang *et al.*, 2022 ▸; Ding *et al.*, 2024*b* ▸), we carried out a principle component analysis of the data set matrix **F** using the SVD 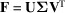, where **U**, 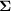 and **V** are matrices of size 6000 × 6000, 6000 × 101 and 101 × 101, respectively. Matrix **V** consists of the singular vectors, and the entries of 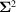 are the corresponding coefficients of the projection of **F** onto the principal vectors in **V**.

**Figure 4:**
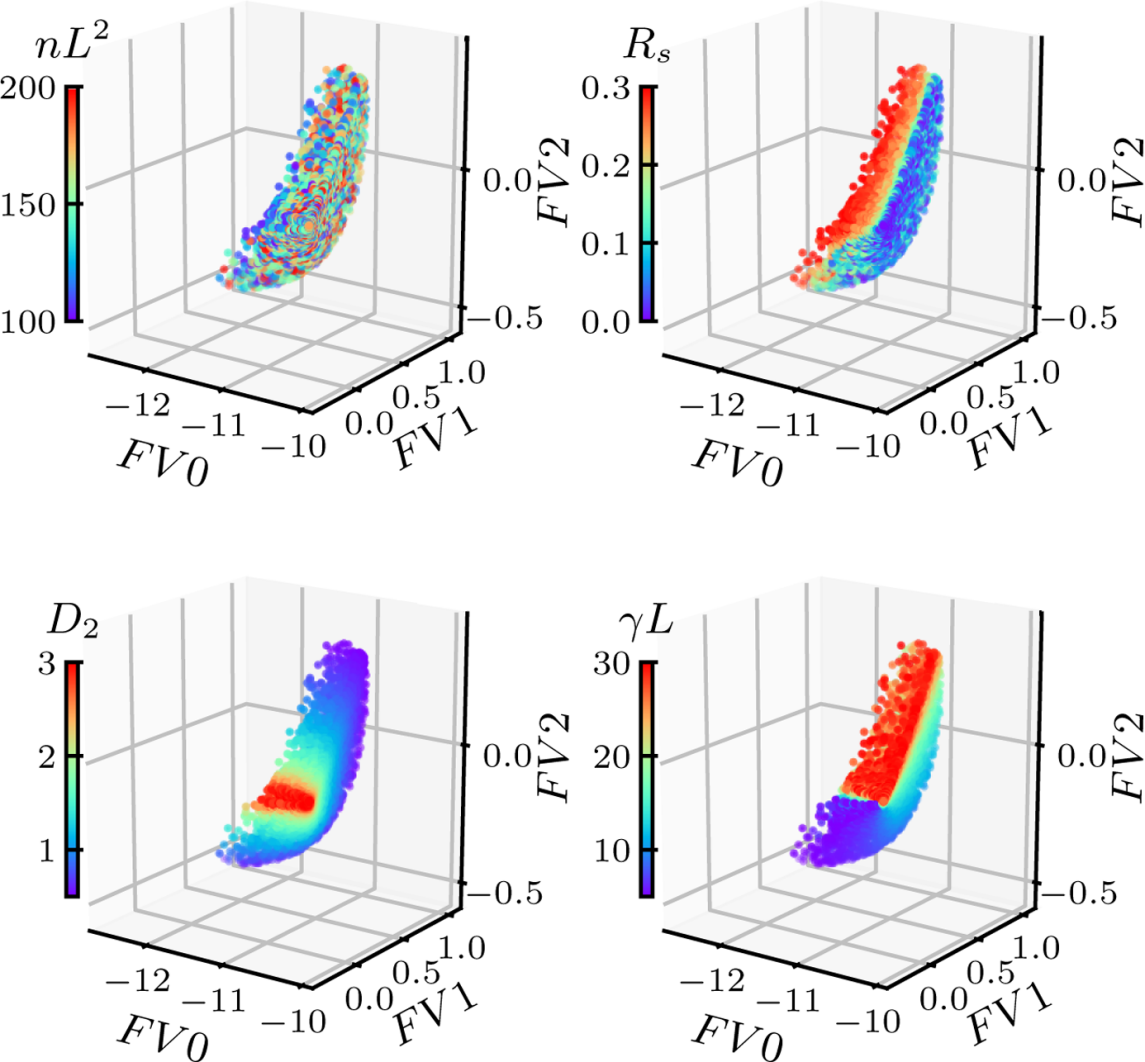
Distribution of the system parameters in the singular value space. (*a*) Number density *nL*^2^. (*b*) Variation of particle size *R*_s_. (*c*) Non-affine transformation *D*_2_. (*d*) Shear rate γ*L*.

Projecting each *g*(θ) in the data set **F** onto the three singular vectors (*V*1, *V*2, *V3*) yields three corresponding projection values (*FV*0, *FV*1, *FV*2), which can be considered as a dimension reduction of the original *g*(θ). This converts each *g*(θ), as well as the system parameter **Y** = {(*nL*^2^, *R*_s_, *D*_2_, γ*L*)} associated with it, in the data set **F** to a point in 3D space spanned by the three projection values (*FV*0, *FV*1, *FV*2). Fig. 4 ▸ shows the distribution of (*nL*^2^, *R*_s_, *D*_2_, γ*L*) in such a space. The distribution shows the feasibility of mapping the features of *g*(θ) back to these system parameters. From the color distribution, we note that the values of (*R*_s_, *D*_2_, γ*L*) are well spread out in the (*FV*0, *FV*1, *FV*2) space, indicating a smooth and continuous mapping from the projection values of *g*(θ) to these corresponding system parameters. However, the distribution of number density *nL*^2^ in (*FV*0, *FV*1, *FV*2) is rather random, implying it is not suitable for the inverted mapping. The inability to extract the number density information from our scattering correlation function is not surprising, as we are working in the limit of dilute dispersions where the number density of particles does not play a role in the microscopic structure or the rearrangement.

### 3.3. Inference of the system parameters

For the ML inversion of system parameters (*R*_s_, *D*_2_, γ*L*) from angular scattering correlation function *g*(θ), we split the data set **F** = {*g*(θ)} into two groups, a training set **F**_train_ = {*g*(θ)}_train _ consisting of 70% of **F**, and a test set **F**_test_ = {*g*(θ)}_test_ consisting of the remaining 30%. We used the training set to optimize the GPR, especially the hyperparameters (*l*, σ) for each system parameter individually by maximizing the log marginal likelihood using gradient descent (Williams & Rasmussen, 2006 ▸). We then used the trained GPR to predict the system parameters of the test set and compare the GPR predicted system parameters with those actually used for the MC simulations.

**Figure 5:**
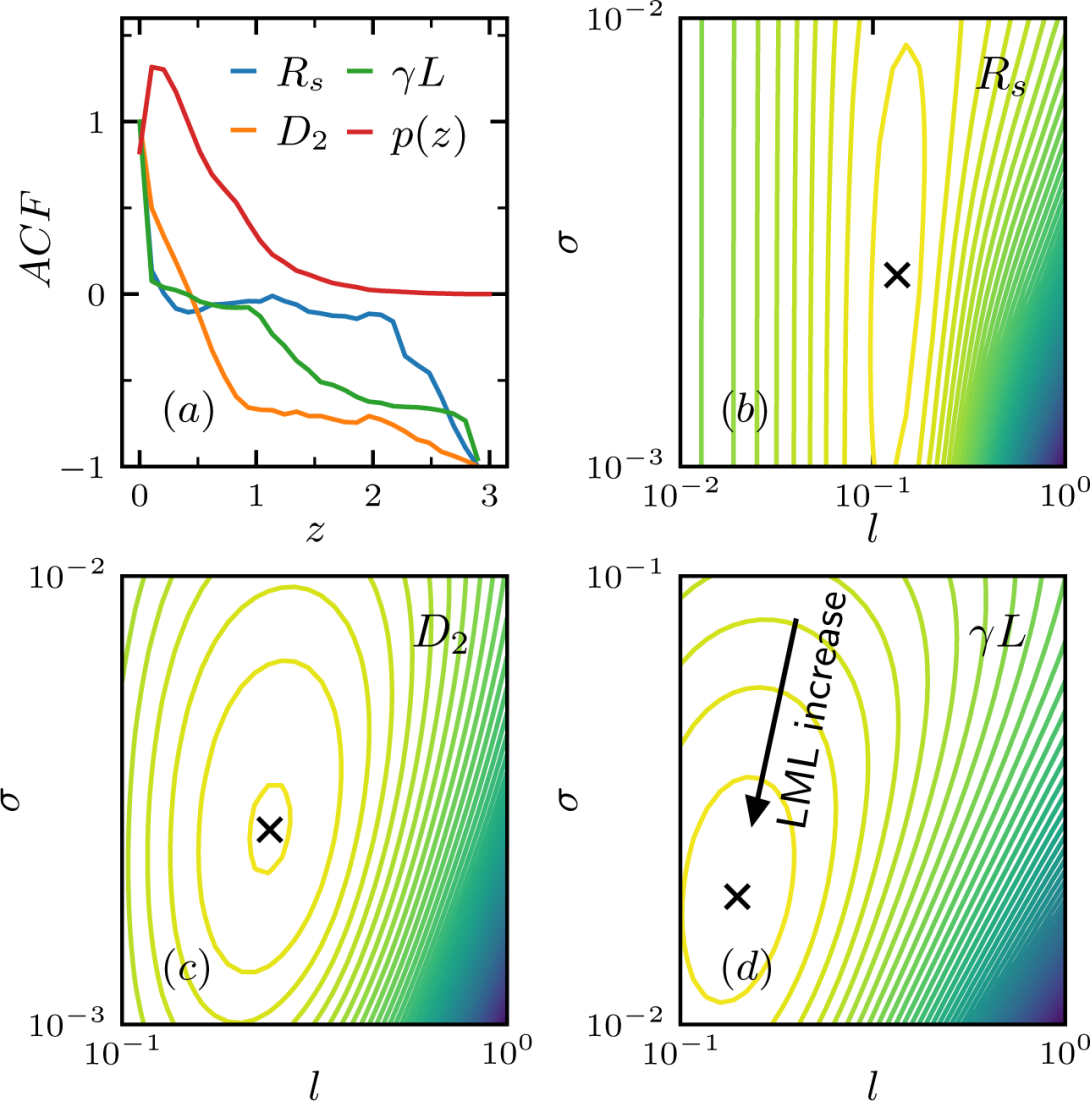
Determining the hyperparameters *l* and σ for system parameters. (*a*) PDDF *p*(*z*) of the data set **F** and ACF for three system parameters. Log marginal likelihood of hyperparameters for system parameters: (*b*) *R*_s_, (*c*) non-affine rearrangement *D*_2_ and (*d*) affine shear strain γ*L*.

Fig. 5 ▸ shows the determination of the log marginal likelihood contour in the (*l*, σ) space for each system parameter (*R*_s_, *D*_2_, γ*L*). To gauge the appropriate range of *l* for each system parameter, as shown in Fig. 5 ▸(*a*), we first analyzed the PDDF of 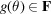 and then investigated the ACF for (*R*_s_, *D*_2_, γ*L*), which gave us a rough range in which we could search for the optimized *l*. The resulting log marginal likelihood contours are shown in Figs. 5 ▸(*b*)–5 ▸(*d*), and the values of optimized (*l*, σ) are shown in Table 1 ▸.

**Figure 6:**
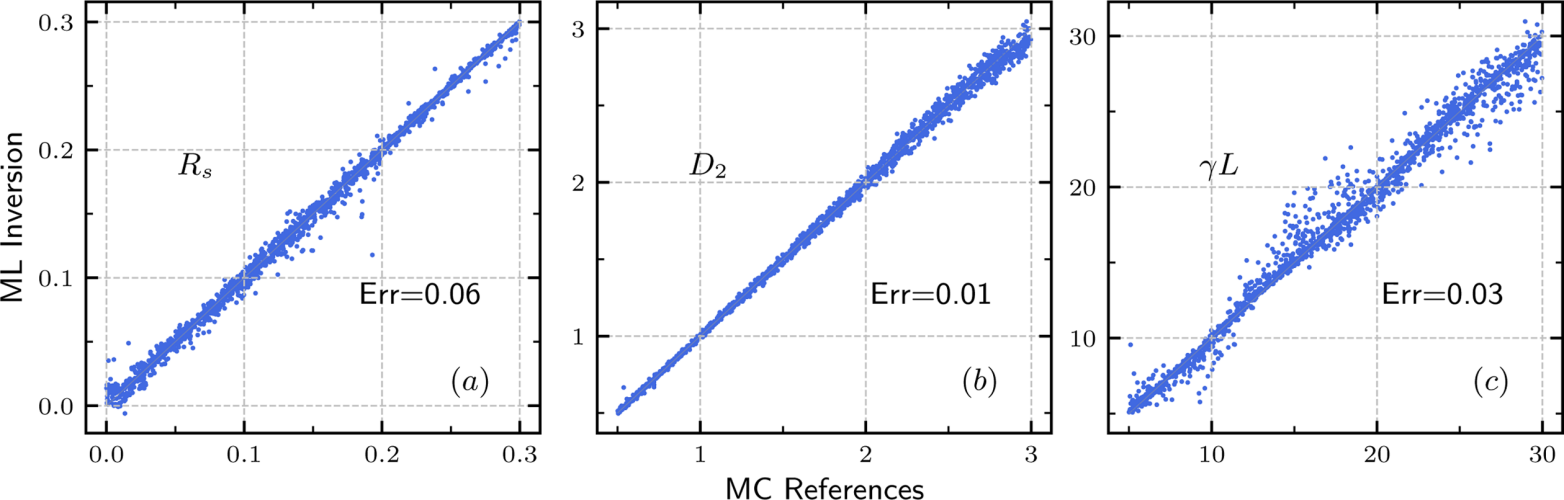
Comparison between the system parameters extracted from the angular scattering correlation function *g*(θ) using the GPR and their corresponding MC reference used for generating scattering data. Averaged relative error Err is indicated in each plot. (*a*) Variation of particles size *R*_s_. (*b*) Non-affine rearrangement *D*_2_. (*c*) Affine shear strain γ*L*.

We applied the trained GPR with the optimized hyperparameters on the test set **F**_test_ to infer ML inverted system parameters (*R*_s_, *D*_2_, γ*L*) and compared the inferred results with MC references. Fig. 6 ▸ shows the comparison of system parameters (*R*_s_, *D*_2_, γ*L*). Almost all of the data points lie around the diagonal line, indicating a good estimation. For each system parameter μ, the relative error between the MC reference μ_MC_ and ML inversion μ_ML_ is estimated by 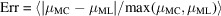, where 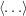 here is averaged over all data points. The relative error is labeled for each system parameter in each panel of Fig. 6 ▸ and shows a very high precision: 1% for *D*_2_, 3% for γ*L* and 6% for *R*_s_. The precise quantification of system parameters demonstrates the power of our ML approach for analyzing and extracting microscopic rearrangement from coherent scattering data.

## 4. Summary

We have presented an ML-informed analysis framework that successfully recovers the polydispersity and microscopic rearrangements, including both affine simple shear and non-affine transformation, with high precision from the correlation function *g*(**q**) of coherent scattering intensity of the dilute dispersions of spherical particles.

Our simulated colloidal systems and scattering intensity aim to mirror real synchrotron scattering setups, including the beam size, particle size and range of the detectable wavevector; therefore, our SVD features and GPR models can easily be compared and adopted to analyze real experimental data. The direction of affine shear is not necessarily always in the *x* direction for experimental data. However, the direction is easy to identify by the high correlation strip in the *g*(**q**) pattern; one can rotate the scattering data before applying our model. Currently, our approach applies to dilute dispersions of spherical colloids. If the system deviates significantly from these assumptions, such as involving anisotropic particles or nonlinear turbulent flow, our model would require retraining with simulations that capture the corresponding scattering behavior. Additionally, incorporating experimental data sets labeled with known microscopic rearrangements, such as shear-driven viscous laminar fluids (Aime & Cipelletti, 2019 ▸), could further enhance its applicability. By leveraging such data, the model could generalize better to real-world experiments, including those with sparse or incomplete data sets. If the experimental scattering data are taken on a different grid of wavevector **q**, interpolated data can be utilized to feed into our GPR model. Alternatively, we can also use generative models such as Kolmogorov–Arnold networks (Liu *et al.*, 2024*b* ▸; Liu *et al.*, 2024*a* ▸) to obtain *I*(**q**) as a continuous function of the system parameters and use it to directly fit the experimental data.

Further, similar methods can be deployed to study the microscopic rearrangement of disordered colloidal systems, like glasses and gels, where ML-assisted quantification methods have a great potential to overcome the challenges imposed by the out-of-equilibrium nature (Schoenholz *et al.*, 2016 ▸; Horwath *et al.*, 2024 ▸). However, for such highly concentrated systems, the MC direct sampling method often suffers from a high rejection rate. As a result, alternative techniques like Brownian dynamics or molecular dynamics simulations are better suited for capturing the intricate dynamics and interactions in these systems.

## Figures and Tables

**Figure 7:**
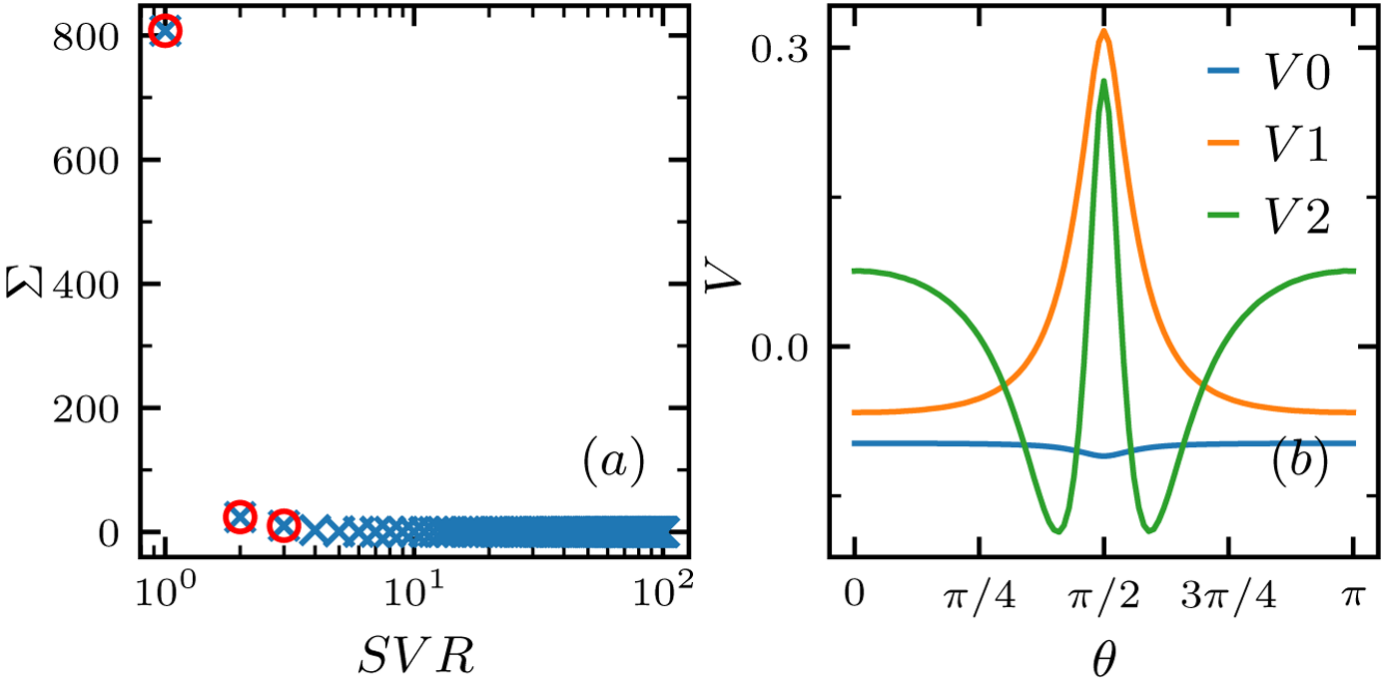
SVD of the angular scattering correlation data set. (*a*) Singular value Σ versus singular value rank (SVR); the top three ranked values are highlighted with red circles. (*b*) Singular vectors corresponding to the first three singular values.

**Table 1:** Optimized hyperparameters for each feature, obtained from the maximum log marginal likelihood

	*l*	σ
*R*_s_	1.338 × 10^−1^	2.673 × 10^−3^
*D*_2_	2.419 × 10^−1^	2.717 × 10^−3^
γ*L*	1.405 × 10^−1^	1.927 × 10^−2^

## Appendix A Singular value decomposition of the data set

Fig. 7 ▸(*a*) shows the singular value versus its rank, where the rapid decay of the singular value indicates that the significance of the projection onto higher-rank singular vectors quickly becomes negligible. Therefore, decomposition of *g*(θ) into the top three singular vectors will provide a good approximation of the whole *g*(θ). Fig. 7 ▸(*b*) shows the singular vectors (*V*1, *V*2, *V*3) corresponding to the first three singular values.
